# How native-like can you possibly get: fMRI evidence for processing accent

**DOI:** 10.3389/fnhum.2015.00587

**Published:** 2015-10-30

**Authors:** Ladan Ghazi-Saidi, Tanya Dash, Ana I. Ansaldo

**Affiliations:** ^1^Centre de Recherche de I’Institut Universitaire de Gériatrie de Montréal, University of MontrealMontreal, QC, Canada; ^2^Faculté de Médecine, Université de MontréalMontreal, QC, Canada

**Keywords:** accent, bilingual, second language acquisition, brain, insula

## Abstract

**Introduction:** If ever attained, adopting native-like accent is achieved late in the learning process. Resemblance between L2 and mother tongue can facilitate L2 learning. In particular, cognates (phonologically and semantically similar words across languages), offer the opportunity to examine the issue of foreign accent in quite a unique manner.

**Methods:** Twelve Spanish speaking (L1) adults learnt French (L2) cognates and practiced their native-like pronunciation by means of a computerized method. After consolidation, they were tested on L1 and L2 oral picture- naming during fMRI scanning.

**Results and Discussion:** The results of the present study show that there is a specific impact of accent on brain activation, even if L2 words are cognates, and belong to a pair of closely related languages. Results point that the insula is a key component of accent processing, which is in line with reports from patients with foreign accent syndrome following damage to the insula (e.g., Katz et al., 2012; Moreno-Torres et al., 2013; Tomasino et al., 2013), and healthy L2 learners (Chee et al., 2004). Thus, the left insula has been consistently related to the integration of attentional and working memory abilities, together with fine-tuning of motor programming to achieve optimal articulation.

## Introduction

Second language (L2) acquisition encompasses mastering many components, including syntax, semantics, pragmatics, phonology, and phonetics. Adopting native-like accent is not always possible, and is mostly a function of age of acquisition (Long, 1990; Bongaerts et al., 1995; Birdsong, 1999; Singleton, 2003). The notion of accent is a complex one, as it concerns a number of features that go from phonological to motor and emotional dimensions. Hyman (2006) describes accent at the word and phrase level, as “stress accent" and “pitch accent" respectively. Moreover, accent is also influences by psychosocial factors, such as cultural background and education. In this regard, Crystal (1987, 2003) have defined accent as the way in which a specific language is pronounced, which allows identifying the region and the social status of the speaker. From a neurolinguistic point of view, accent comprises processing phonology, prosody, intonation, as well as motor programming and planning. Phonetic and prosodic rules that characterize a specific language are crucial features of accent. Thus, accent concerns segmental (i.e., prosodic distinction) and supra-segmental units (i.e., loudness, pitch and duration). Prosodic distinction is considered segmental based on its position in entire prosodic structure (Keating, 2006). For example, the phonetic realization of a consonant /p/ depends on the consonants’ position in the prosodic structure (i.e., where the terminal node is going to come). As it is the case with other domains of language development, accent in mother tongue is acquired automatically (i.e., unconsciously). Hence, as it is acquired naturally as a lexical or a phrasal language component, it is hard to dissociate from language processing as a whole. However, in cases where the native speaker decides to change accent for pragmatic reasons (i.e., to mark social status, political or academic purposes, cultural influences, or in circumstances such as acting or dubbing), conscious control of accent is required, and changing accents may be even effortful.

In the context of second language (L2) learning, accent processing generally necessitates some level of cognitive control, particularly when the age of acquisition is above the critical period (Bongaerts et al., 1995; Birdsong, 1999; Singleton, 2003). Thus, there is evidence that children lose their capacity to perceive and distinguish phonemes as early as 6 months to 12 months of age (Werker and Desjardins, 1995), and therefore, after that critical period, the production of phonemes in L2 will be influenced by the mother tongue. Consequently, it has been argued that foreign accent can be detected both in early and late L2 learners (Barlow, 2014), in particular if L2 has been learnt in a formal manner (Peltola et al., 2003, 2007; Best and Tyler, 2007; Jost et al., 2015). Perhaps this is one of the reasons native-like accent has been used as an indicator of high proficiency in second language. This L2 native-likeness depends on a number of factors, including L2 age of acquisition, frequency of L2 use and amount of exposure, gender, formal training, motivation, amount of continued L1 use (Flege et al., 1995; Piske et al., 2001), as well as type of L2 learning approach and linguistic distance which eventually leads to inaccessible perceptual representation to L2 learners (Flege, 2003). Thus, regarding learning approach, there is evidence that it is more likely to achieve native-like accent when L2 acquisition occurs by informal exposure and interaction in a naturalistic L2 context, especially through interaction with friends, rather than formal training such as taking lessons in a classroom (Polat, 2007). As for the influence of linguistic distance on accent processing, the more the structural similarities across L1 and L2, the larger the overlap at the phonological and phonetic levels (Ringbom, 2007), and thus, the smaller the load on attentional and motor-processing abilities. However, there is evidence of better acquisition of L2 phonemes when the later are not part of the L1 phonology (Best and Tyler, 2007). Interestingly, there are models (PAM: Perceptual Assimilation Model, Best, 1995; SLM: Speech Learning Model, Flege, 1995), which interpret L2 sounds from L1 pre-existing Structures. In contrast, Markedness differential hypothesis (Eckman, 1977) functions on typological markedness between L1 and L2. Grimaldi et al. (2014) by using mismatch negativity experiments of speech sound discrimination confirmed the PAM framework suggesting assimilation of L2 vowels in the pre-existing phonology in absence of L2 phonetic discrimination. Lack of discrimination for vowel contrast was reported by Peltola et al. (2007), in early language immersion programs, in the form of the absence of native like memory trace of L2 vowels. On the other hand, Zsiga (2003) reported an asymmetrical transfer of patterns in L1–L2 syllable sequences thus supporting a default pattern of articulation uncharacteristic of L1 and L2. Such work is in favor to Markedness differential hypothesis, i.e., there are universal contrasts, which favor less marked structures, thus the asymmetry in the transfer between L1 and L2.

The study of accent has been approached by different means. Studies on adult healthy populations, including second-language learners and studies on clinical populations presenting disordered speech motor control or other clinical conditions. Also, different methodologies have been used to explore the neural basis of accent, namely anatomical and functional neuroimaging, as well as computational modeling, based on clinical data.

Different brain regions play crucial role in speech motor production (Guenther, 2006; Guenther et al., 2006). The Directions Into Velocities of Articulators (DiVA) is a computational model, which argues for the role of the insula in articulation, and highlights the similarity between this role and that of the premotor and motor cortices (Guenther et al., 2004). Thus, the DiVA, model includes the insula as part of the motor speech control circuit, and thus, participating to in accent processing.

The neural basis of speech motor control has been extensively approached by means of behavioral and functional neuroimaging tools, but very few of them have specifically focused on the neural basis of accent processing. Specifically with healthy populations, one ERP study (Romero-Rivas et al., 2015), and one fMRI study on the comprehension of foreign accent speech (Adank et al., 2012) have been published. However, there are no neuroimaging studies on the production of accent in healthy population. Most data on accent processing comes from clinical reports, some of which include a comparison of functional neuroimaging in healthy control participants and patients with foreign accent syndrome (FAS), (Fridriksson et al., 2005; Poulin et al., 2007; Katz et al., 2012; Moreno-Torres et al., 2013; Tomasino et al., 2013) or AOS (Moser et al., 2009). Other case reports include imaging reports on a variety of brain- damaged populations presenting speech disorders (e.g., Kent and Rosenbek, 1983; Wertz et al., 1984; Gurd and Coleman, 2006; Mariën and Verhoeven, 2007; Moreno-Torres et al., 2013; Tomasino et al., 2013). The next paragraphs develop on these accounts. Motor speech disorders include apraxia of speech (AOS), Dysarthria and FAS, all of which are characterized by the disruption of phonetic-prosodic components of speech production, affecting the naturalness and native-likeness of speech. Specifically, AOS is characterized by an impaired ability of initiation, sequencing, timing, coordination and vocal tract shaping for speech sound production (Kent and Rosenbek, 1983; Wertz et al., 1984), and disrupted fine-tuning of the balance between production of phonetics and prosodic units of speech (Boutsen and Christman, 2002; Aichert and Ziegler, 2004) resulting in unnatural production of speech sound. This accent pattern has been related to damage in the left inferior frontal gyrus, and the anterior insula, both areas having been reported to play a role in novel speech production, particularly in regard to the facilitation of new motor plans for speech (Chee et al., 2004). On the same line, Moser et al. (2009) conducted an fMRI study with 30 healthy adults on a non-word-repetition task with English (native) or Non-English (novel) syllables; the authors (Moser et al., 2009) found greater activation in anterior insula with a novel syllable processing as compared to native syllable sequences. A single-case study Hiraga et al. (2010), provides further evidence on the role of the insula in accent processing, this time in the context of pure dysarthria. Dysarthria is characterized by deficient articulation resulting from reduced motor strength and motor coordination, and/or to defects of the articulatory apparatus (Goodglass, 1993). In their single-case study, the authors (Hiraga et al., 2010) reported on a 72-year-old male who suffered posterior insular damage with dysarthria and no aphasia. Although limited to a single-case, this study points to the role of the insula in the processing of accent.

Studies on FAS have been very important in pointing to the role of specific networks in the processing of accent. FAS is characterized by pronunciation alternations that make the native speaker of a given language sound as a foreign speaker. These alternations, at least in English, include syllable-timed speech rhythm instead of stress-timed speech rhythm, the insertion of epenthetic vowels, that change syllable structure, tense vowel systems in place of tense/lax systems, and sentential intonation, patterns with rising contours (Blumstein and Kurowski, 2006). This condition does not include phonological errors, and it is distinct in both its characteristics and underlying mechanisms from an AOS, a dysarthria and an aphasic speech output (Blumstein and Kurowski, 2006). Berthier et al. (1991), discuss four cases of FAS, by reference to all 10 case reports since 1919 and concluded that for the disorder to be diagnosed as FAS, co-occurrence of segmental and prosodic deficits is essential. They associate FAS to damage in the precentral gyrus, with better recovery being observed following premotor damage. The collective evidences across a variety of clinical populations shows the role of a set of areas in accent processing; these include Broca’s area (frontal operculum and posterior third of the inferior frontal gyrus), the premotor cortex, the striatum, the insula, the pallidum, the thalamus, as well as white-matter pathways of the internal capsule—all typically on the left side in right-handed patients (Kurowski et al., 1996; Gurd and Coleman, 2006; Mariën et al., 2006, 2009; Scott et al., 2006; Kuschmann et al., 2012).

Functional neuroimaging evidence from FAS comes from comparisons between FAS patients and controls, performing a variety of language tasks (Fridriksson et al., 2005; Poulin et al., 2007; Katz et al., 2012; Moreno-Torres et al., 2013; Tomasino et al., 2013). Moreno-Torres et al. (2013) reported on a middle-aged bilingual woman with chronic FAS, characterized by deficits including changes in linguistic and emotional prosody, as well as lack of motivation to communicate. Magnetic resonance imaging (MRI) showed bilateral lesions, particularly in the left deep frontal operculum, and dorsal anterior insula. Also, Diffusion tensor Imaging (DTI) and Tractography suggested disrupted left deep frontal operculum-anterior insula connectivity. Positron emission tomography (PET) showed decreased activation in Brodmann’s areas 4, 6, 9, 10, 13, 25, 47, in the basal ganglia, and anterior cerebellar vermis. The authors (Moreno-Torres et al., 2013) argue that the ensemble of the neurofunctional and neuroanatomical evidence from this single-case report suggests that FAS entails altered planning and execution of speech production, with both cognitive control and emotional communication dimensions. Moreover, this report shows the key role played by the insula-frontal operculum circuit in the processing of accent. In another study using functional MRI, Katz et al. (2012) reported the activation maps related to a picture-naming task in an English-speaking woman with FAS of unknown etiology. The activations included the left superior temporal and medial frontal structures, bilateral subcortical structures and thalamus, the left insula and the left cerebellum. Similarly, in their PET study, Tomasino et al. (2013) compared the accent of a patient suffering from FSA secondary to damage to the putamen, to that of a group of healthy controls, in the context of counting, sentence and pseudoword production and picture naming. As compared to healthy subjects, the patient showed an increased activation in the pre/postcentral gyrus and ventral angular gyrus. Authors conclude that FAS is a result of an impairment of the feed-forward control commands, in particular of the articulator velocity and position maps (Tomasino et al., 2013). Another PET study by Poulin et al. (2007) examined FAS in a case of bipolar syndrome and reported hypometabolism in the frontal, parietal and temporal lobes bilaterally, as well as a focal damage in the left insular and anterior temporal cortex (Poulin et al., 2007), thus pointing to the role of the anterior temporal gyrus and the left insula in accent processing. Finally, Fridriksson et al. (2005), report the case of a stroke patient with damage in the putamen and extending fiber tracts, showing symptoms of FSA. Concurrently with impaired motor speech regulation, fMRI results with an overt picture-naming task show a significant activation of the superior temporal and inferior frontal lobes, as well as in the inferior motor strip (face region) and the lateral occipital gyri. The authors (Fridriksson et al., 2005) argued that the lesion resulted in apraxia and FAS symptoms as a consequence of increased reliance on motor execution, as reflected by the activation motor cortex (Fridriksson et al., 2005). Another possible interpretation is that damage to the fiber tracts disconnected this circuit from the insula and leading to the reported FAS symptoms.

Despite the interest of the previous studies, it is difficult to draw any strong conclusions regarding the activation patterns reported in regard to the neural basis of accent. Thus, the activation maps observed in these patients are not exclusive to accent processing, but reflect a variety of task processing components. Also, given that brain damage disrupts complex brain circuits, and leads to symptoms that reflect both damage and compensation to damage, it is not possible to draw conclusions regarding the areas or set of areas specifically related to accent processing. In this regard studies with healthy and in particular, studies with second language learners, could open a window onto the normal neural mechanisms underlying the production of a foreign accent. In particular, fMRI studies on cognate learning in healthy adults can shed light on the neural basis of accent processing. Thus, cognates share phonological and semantic features across languages, and thus they are easier and faster to learn than non-cognates, which share semantics only, and clangs which share phonology but not semantics (De Groot, 1992, 1993; Sánchez-Casas et al., 1992; Ellis and Beaton, 1993; Kroll and Stewart, 1994; De Groot and Keijzer, 2000; Hall, 2002; Sánchez-Casas et al., 2005; Christoffels et al., 2007). Moreover, when learning of cognate is consolidated, they are almost processed as mother tongue (Perani et al., 1996; De Bleser et al., 2003). Still, there are subtle differences in the pronunciation of cognates at the level of intonation, prosody, and articulation placement lead to what we perceive as accent, which make cognates good candidates to isolate the neural markers of foreign accent in the healthy brain.

In the present study, we examined 12 healthy Spanish-speaking adults, who learnt French cognates by means of a computerized training program. They were trained for 4 weeks, until they attained a perfect score in picture naming of L2 cognates, after which they were tested on picture naming of cognates during fMRI scanning, both in L2 and their mother tongue.

## Materials and Methods

### Experimental Design

This was an event related fMRI group study on cognate naming. Behavioral and event-related fMRI measures were collected after 4 weeks of cognate naming training, when participants attained a 100% success rate in naming. The study included a pre-experimental assessment on bilingualism and cognitive status, and the completion of a computerized vocabulary-learning program. Participants received a short training on the use of the program, followed by self-training, for 15 min a day during 30 days. When participants attained 100% success rate on naming, they were tested on naming the object pictures in L2 (French), and in their mother tongue (Spanish). Accuracy rates (ARs), accent judgment rating and RT were collected, and activation maps relative to event-related BOLD responses were extracted. More details about participants, stimuli, training program and the task will follow.

### Participants

Twelve Spanish-speaking (L1) adults (40.7 ± 13.0 years, range 26–66; six males, six females), with no history of neurological or neuropsychological disorders, participated in our study (refer to **Table 1** for details). All participants were right-handed, as measured by the Edinburgh Handedness Inventory (Oldfield, 1971), and were homogeneous in terms of their educational background (15.8 ± 1.5 years, range 12–18), and were matched for an elementary level of French from the elementary level immersion courses offered by the Quebec government for immigrants, who were tested to have no knowledge of French by a placement test. Given that students pass standardized placement tests and a thorough interview to be admitted to these courses, this ensured an equal amount of exposure to L2 at recruitment and an equivalent level of L2 knowledge. In addition, baseline in L2 proficiency was determined by means of a questionnaire based on the work of Paradis and Libben (1987), Flege (1999), Silverberg and Samuel (2004) used in previous studies in our laboratory (Raboyeau et al., 2010; Scherer et al., 2012; Marcotte and Ansaldo, 2014). This modified version of the questionnaire gives information on the participants’ level of proficiency, L2 language use as well as the duration of the L2 courses (refer to **Table 2** for details). All the participants reported of minimal exposure of French at home as well as outside home. The participants were highly motivated toward the French language course as means of successful participation in the different social settings/community through the use of language. Further, participants were tested on their knowledge of the experimental stimuli before they experienced any lexical learning in L2; being able to name more than 15% of the stimuli, thus five stimuli, was considered an exclusion criteria.

**Table 1:** Participants’ demographic data and Neuropsychological test results including: Montreal Cognitive Assessment (MOCA) Memory test (Nasreddine et al., 2005); Memory and Learning Test (Grober and Buschke, 1987; Grober et al., 1988), and the Attention and inhibition Stroop test (Beauchemin et al., 1996).

Participants	Gender	Age (months)	Years of education (Years)
1	f	38	16
2	m	32	16
3	f	26	14
4	f	54	16
5	f	52	16
6	m	36	16
7	m	26	18
8	m	38	18
9	f	28	16
10	f	37	16
11	m	58	16
12	m	64	12
***M***	6 m	40.75	15.8
***SD***	6 f	13	1.5

**Table 2:** Information on the participants’ knowledge of L2 (French) at baseline.

Participants’ L2 Knowledge (out of ten participants participating in the study)	
Considers his ability to communicate in French limited	7
Considers L1 accent when speaking in French evident	11
Considers himself moderately understandable in French	10
Rated below average level of production in French on Québec Ministry of Education tests	9
Rated below average level of comprehension in French on Québec Ministry of Education tests	9
Rated below average level of reading in French on Québec Ministry of Education tests	10
Rated below average level of writing in French on Québec Ministry of Education tests	10

Cognitive factors that may have an influence on L2 vocabulary learning were controlled by means of a series of tests. Mild cognitive impairment was ruled out by means of the Montreal Cognitive Assessment (MOCA, Nasreddine et al., 2005). Only participants with score of 26 or more (29.8 ± 0.77) were included. The Memory and Learning Test (Grober and Buschke, 1987; Grober et al., 1988) controlled for memory and learning skills, participants free recall skills (27.25 ± 4.24 raw score) and category recall skills (9.9 ± 2.0 raw score), and the non-verbal Stroop test (Beauchemin et al., 1996) for attentional and executive function abilities color-time (17.6 ± 6.01 s), word-time (16.2 ± 5.6 s) and word-color (22.3 ± 6.7 s). Participants who performed above cut-off levels for this battery were recruited to participate. After completing the pre-experimental assessment, participants were enrolled in a computerized lexical-training program in French.

#### Stimuli

The experimental list included 35 Spanish-French Cognates (*N* = 35; e.g., Téléphone /telefOn/, French, and Teléfono /telefOnO/, Spanish; both words meaning ‘telephone’), and a set of color photographs depicting each of them (refer to **Table 3** for complete list of stimuli). Stimuli were matched across languages for: visual complexity, object and word familiarity, lexical frequency, word length, and number of phonemes and syllables. An equal number of items were selected for animals, fruits and vegetables, clothes and accessories, stationery, and household objects to control for a possible category effect (Caramazza and Shelton, 1998). Twenty distorted images were used as the control condition for fMRI scanning.

**Table 3:** List of Spanish–French cognates.

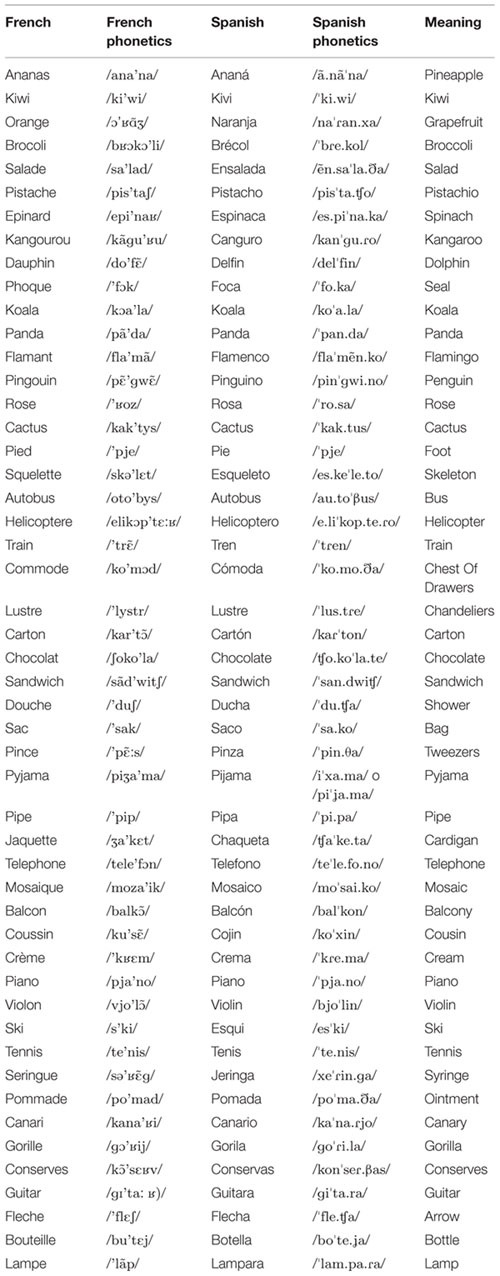

#### Lexical Training Program

Similar to our previous studies (Raboyeau et al., 2010; Ghazi-Saidi et al., 2013), participants completed a computerized self-training to learn 35 cognates and their native-like pronunciation in Canadian French (Québécois). Participants spent 15 min a day during 4 weeks. With the computer software, the target picture is displayed on the screen, followed by a series of phonological cues, displayed under the target picture when an icon is pressed. The first cue is the first sound of the target word, followed by the first and second sounds, and finally the whole target word. Participants were instructed to look at the picture, and listen to the first cue, then to the second cue, and then to the whole word. They were allowed to repeat this procedure as many times as necessary to learn the word. In their subsequent practice sessions, participants would first try to name the object when they saw the target picture; if unsuccessful, they would press on the icon and listen to the first cue; if they failed to recall the name of the object, they would listen to the second cue, and if necessary to the whole word. Participants were asked to make an effort to pronounce the word as similarly as possible to the native pronunciation as possible. Thorough instructions were given to the participants at the beginning of the experiment; the respect of all instructions was checked with each participant, on the phone and by e-mail every 2–3 days. Participants were fully committed to respecting the 15-min training routine.

#### Experimental Task and Procedure

After the consolidation, participants were tested on an overt picture-naming task during fMRI scanning. Task instruction was to look at colorful photos of objects and name the object and pronounce the target word as closely as possible to the French native model they have been practicing, and to say dido (a pseudo-word in Spanish, French, and English, in response to seeing distorted images). The task was performed both in L2 (French) and in L1 (Spanish). The event-related experimental design included two runs. Thus, in Run 1, participants were asked to name the cognate pictures in L2, and to do so as closely as possible to the native accent, whereas in Run 2, they were asked to name the same pictures, in their mother tongue. The procedure and task were practiced in the fMRI simulator for optimal data acquisition conditions in the fMRI scanner. Stimuli were displayed by means of a computer equipped with Presentation software v.11.2^1^ Participants lay on their back with their head fixed by cushions and belts, and an fMRI-compatible microphone (MRConfon Optical microphone) was placed close to the participant’s mouth to record responses. No bite-bars were used to allow accurate articulation and also considering that the evidence does not support the use of this device, as it may add extra inconveniences for the participants and thus affect their attention and performance (Heim et al., 2006). Rigid-body head movements were corrected with online movement correction. Before the naming task, and as practiced in the simulator, participants were instructed to look at the computer screen and name aloud each of the pictures presented to the as accurately and as quickly as possible. These pictures were the same as those used in the training phase (*N* = 35 stimuli) presented randomly by means of Presentation v11.2. Each picture was presented for 4 s, after which there would be a blank page for a randomized interval of 4600–8600 ms, then the next picture would be presented. Oral responses were acquired with the fMRI-compatible microphone, and Sound Forge software (Sonic Foundry, Madison, WI, USA). Following our previous studies (Raboyeau et al., 2010; Ghazi-Saidi et al., 2013), we used a variable inter-stimulus interval (ISI) to assure a better sampling of the hemodynamic response and prevent attentional bias (Huettel et al., 2004).

#### fMRI Parameters

Acquisition parameters were the same as in previous studies in our laboratory (Raboyeau et al., 2010; Ghazi-Saidi et al., 2013). The acquisition included 28 slides in the axial plane, so as to scan the whole brain, including the cerebellum. Sequential slices were collected, to avoid the stripping that might happen because of certain types of head motion (Siemens 3T Scanner User Training: Supporting Information and FAQ).

Stimulus presentation time was 4500 ms, with a variable ISI (between 4325 and 8375 ms), TR = 3 s, TE = 40 ms, matrix = 64 × 64 voxels, FOV = 24 cm, and slice thick- ness = 5 mm. A high-resolution structural scan was obtained during the two functional runs (naming in L1 and naming in L2), using a 3D T1-weighted pulse sequence MPRAGE (TR = 2.3 ms, TE = 4.92 ms, angle = 25°, 76 slices, matrix = 256 × 256 mm, size = 1 mm × 1 mm × 1 mm, FOV = 28 cm).

#### Ethical Issues

This study was approved by the ethics committee of Réseau de Neuroimagerie du Québec (RNQ). All participants signed a consent form. The procedure was explained clearly to the participants. All data were recorded in the Unité neuroimagerie fonctionnelle (UNF) at the Institut de Gériatrie de Montréal (IUGM). Appendix 1 includes the UNF screening form and can be found in the online version, at http://dx.doi.org/10.1016/j.bandl.2012.11.008.

### Data Analysis

#### Demographic and L2 Knowledge Related Patterns

A jury of three Canadian French native speakers rated the degree of native-likeness of word pronunciations following cognate learning. Raters included two women and four men, aged between 22 and 48. All raters were born and raised in Quebec, and were native speakers of Canadian French. Raters were asked to answer a questionnaire on their demographic information and their French knowledge. However, only three raters (two men and one woman) provided a complete rating of the accent characteristics of the participants, the three others were excluded, as they had not rated all of the stimuli, or had found it difficult to listen to some of the recordings.

The rating procedure was based on the procedure used by Piske et al. (2001). Thus, all naming responses in L2 were recorded for each participant. Each rater was given a scale (see Appendix 2) and asked to listen to responses and rate how native-like each participant’s accent. The instruction read as follows: please circle the value that you give to each participant, on the scale. On this scale 1 is very foreign and 9 is native-like. Raters rated each participant individually on a scale of 1–9, for one having heavy foreign accent and nine being perceived as a Canadian French native speaker. Please see Appendix 2. Questionnaire and the scale filled up by Canadian French Native raters.

### Behavioral Data Analysis

The event-related design allowed discriminating between correct and incorrect responses. Response times (RTs) and ARs were calculated. Non-responses, Spanish words, and phonological errors (e.g., /pi/ instead of /pie/) were considered wrong answers, and thus not included in further analysis. Statistical analysis included ARs and RTs for Cognates as well as the pseudo-word with SPSS, version 17.0.

### Neuroimaging Data Analysis

BOLD responses were analyzed for the correctly named items following the data analysis plan of our previous work (Raboyeau et al., 2010; Ghazi-Saidi et al., 2013; Marcotte and Ansaldo, 2014). Neuroimaging data was analyzed with Statistical Parametric Mapping-8 *(SPM-8, Welcome Trust Centre for Neuroimaging, Department of Cognitive Neurology, London, UK)*, established in Matlab *(MathworksInc, Sherborn, MA, USA)*^2^ Data analysis was performed individually first, and them within the group of participants. Slice timing, realignment, normalization, and segmentation were performed first. Images were spatially smoothed with an 8-mm Gaussian filter. Only BOLD responses for correctly retrieved words were included in the analysis. For each participant and for the whole group, task-related BOLD changes were examined by a convolving vector of the onset of the stimuli with a hemodynamic response function (HRF), and its temporal derivative. Statistical parametric maps were obtained for each individual subject, by applying linear contrasts to the parameter estimates for the events of interest (the correct responses); this resulted in a t-statistic map for every voxel.

One-sample *t*-test, group averages were calculated for Cognates minus the control condition (i.e., Cognates –dido). Cluster size (*k*) was superior to 20 voxels and *p* < 0.001. Further, direct contrasts were performed to examine the neural substrate that characterized the processing of accent, with the contrasts: (CognateL2 vs. CognateL1), Significant activated clusters were considered were larger than 15 voxels (*k* > 15) and *p*-value was settled at 0.001.

## Results

### Behavioral Results with Cognate Learning

Mean ARs for naming cognates in L2 (*M* = 85.9, *SD* = 1.4). Correct responses for naming L2 Cognates, in the scanner, included an average of 30 items (maximum = 33, minimum = 28). Further, there was no significant difference in the RTs (in seconds) for naming Cognates in L2 (*M* = 1.81, *SD* = 0.64) and Cognates in L1 (*M* = 1.61, *SD* = 0.4); *t* (0.93) = 0.21, *p* = 0.36.

#### Accent Analysis Results

The jury of native speakers (raters) considered that participants showed a heavy foreign accent when producing learnt cognates (*M* = 3.1; *SD* = 1.4, on a scale of one to nine, where a score of 1 corresponds to perception of a strong foreign accent, and a score of 9 corresponds to the perception of Native). Amount of agreement between the three raters was 52% (κ = 0.346), indicating fair agreement (Landis and Koch, 1977).

### Neuroimaging Results

The fMRI contrast between L2 Cognates and Dido (i.e., Cognate L2 – Dido) for naming images in L2 (French), shoed a significant activation in the left Middle occipital gyrus, the left Lingual gyrus, the left Inferior frontal gyrus, the left Precentral gyrus, the left Inferior frontal gyrus and the left, and the right Middle occipital gyri, the right Parahippocampal gyrus and the right Cerebellar tonsil.

T-contrast fMRI analysis (i.e., Cognates L2-Cognates L1) showed a single significant activation, located in the left Insula.

**FIGURE 1:**
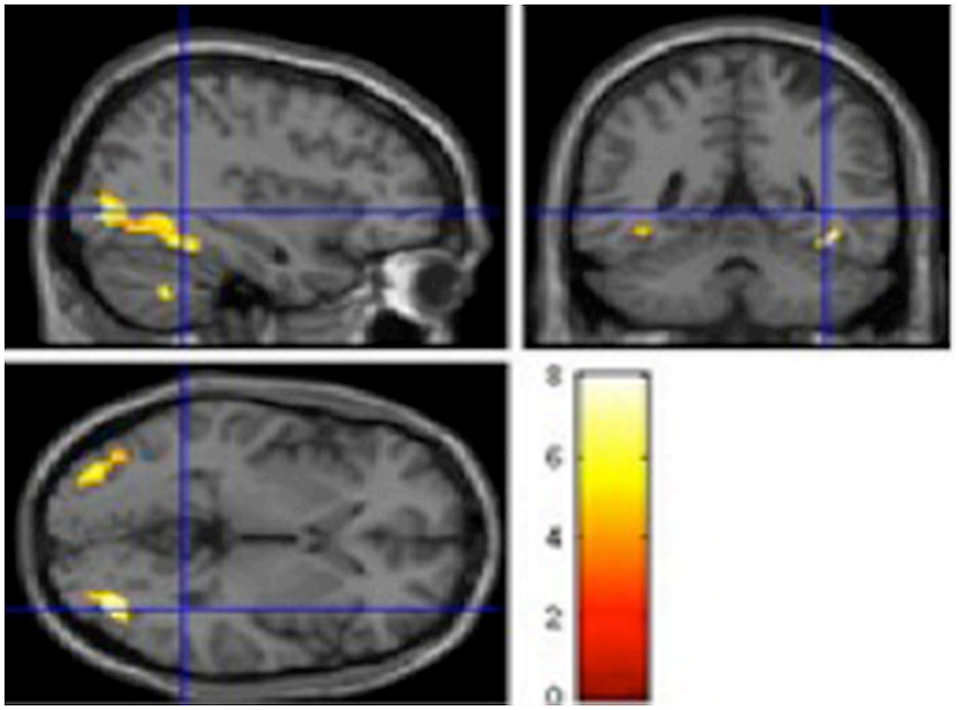
**Significant BOLD signal increase (cluster size (k) superior to 20 voxels and *p* < 0.001), related to Simple contrasts with naming Cognates activated the left Middle occipital and Lingual gyrus (BA37 and BA19), the Inferior frontal gyrus (BA 46 and BA 47) and the left Precentral gyrus (BA6 and BA9), the right Middle occipital gyrus and the righ Parahippocampal gyrus (BA19) and the right cerebellum).** Statistical parametric maps overlaid onto the average T1-weighted anatomy of all subjects (*n* = 12). Activation related to only one layer is presented, thus many activations may not be seen on this image.

**FIGURE 2:**
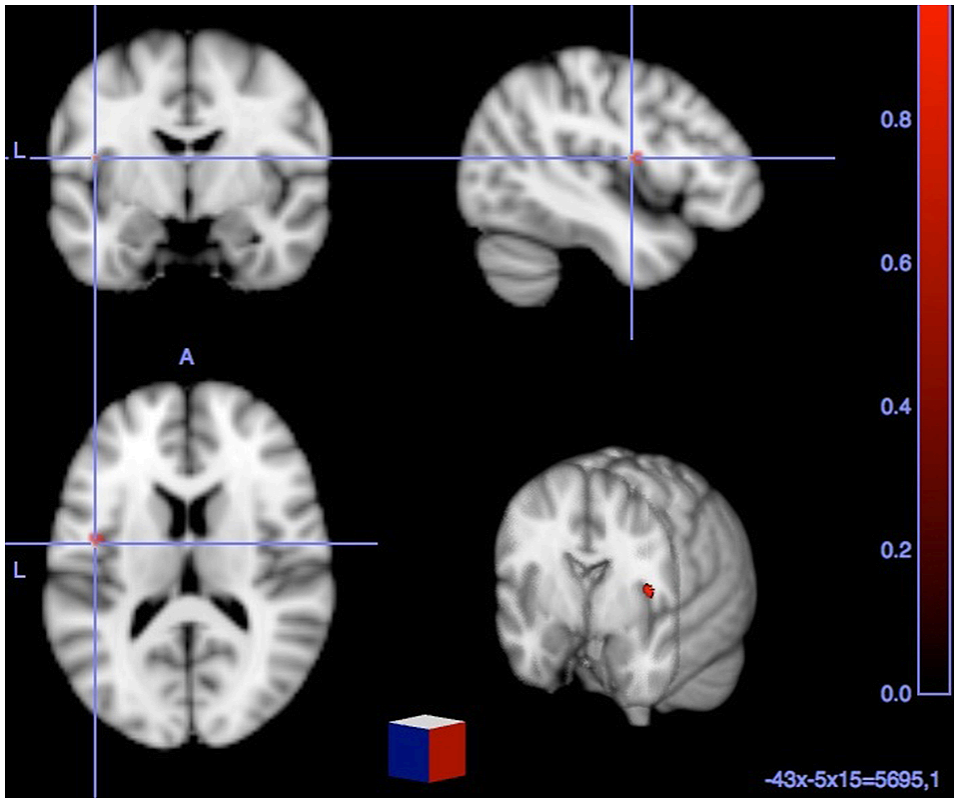
**Significant BOLD signal increase [cluster size (*k*) superior to 15 voxels and *p* < 0.001] related to direct comparisons between each (Cognates – Non-cognates) and (Cognates L2 – Cognates L1) yielded to significant activation in the left insula (BA 44)**.

**Table 4** summarizes the details of these activations and **Figures 1** and **2** show these activations.

**Table 4:** T-contrast fMRI analysis of Cognates L2 vs. Control condition (i.e., Cognates L2 - dido), and the direct contrasts of Cognates (i.e., Cognates L2-Cognates L1), (*k* > 15, *p* < 0.001).

Left							Right						
Regions	*BA*	*x*	*y*	*z*	*T-score*	*Cluster size*	*Regions*	*BA*	*x*	*y*	*z*	*T-score*	*Cluster size*
***Cognates – Dido***												
Sub-gyral/Middle occipital/Lingual gyrus	37/19	-44	-42	-14	7.94	356	Middle occipital gyrus/Parahippocampal gyrus	19	38	-82	-2	8.05	458
Inferior frontal gyrus/Precentralgyrus	9/6	-44	8	24	4.77	95	Cerebellar tonsil		36	-58	-40	6.60	36
Inferior frontal gyrus	45/46	-44	32	-12	4.77	77							
Precentralgyrus	9	-38	4	42	4.68	26							
**Cognates L2 – Cognates L1**												
Insula	13	-44	3	13	3.73	15							

*Significant activated areas resulting from comparing naming Cognates L2 (French) and the control condition (dido), (k > 15, p < 0.001). BOLD responses yielded in activation of the left Middle occipital gyrus, the left Lingual gyrus, the left Inferior frontal gyrus, the left Precentralgyrus, the left Inferior frontal gyrus and the left, the right Middle occipital gyrus, the right Parahippocampalgyrus and the right Cerebellar tonsil. T-contrast fMRI analysis (i.e., Cognates L2-Cognates L1) showed a single significant activation, located in the left Insula.*

## Discussion

Studies on accent processing have mostly focused on clinical populations, with a variety of clinical conditions. The evidence from these studies provides some important clues regarding the neurobiology of accent. However, the complexity of the clinical conditions, the variety of lesion types, sizes and methods used to examine those cases precludes any generalization regarding the neural basis of accent. Moreover, given that clinical signs may reflect not only the effect of damage, but also compensatory mechanisms put into play after damage, and considering that most of this literature concerns single case reports, the results of this research require parsimonious interpretation.

The neural basis of accent processing can be further examined by looking at healthy populations learning a second language, in particular, learning cognates, words that share phonological and semantic features across languages, but still offer the opportunity to examine the impact of accent related differences, at the segmental or suprasegmental levels. In the present study, we examined a group of Spanish speaking (L1) adults learnt French (L2) cognates by means of an audio-visual computerized method; after consolidating cognate picture naming, thus when participants attained maximum score on picture naming of cognates, a test on L1 and L2 oral cognate naming during fMRI scanning was performed. Participants were instructed to respect native accent in each language as much as possible.

Behavioral results showed that mean ARs and RTs did not differ across L1 and L2, which suggests consolidated learning of L2 cognates. However, a jury of native speakers perceived participants’ L2 accent as foreign, as rated on a scale of 1–9, where nine being perceived as a Canadian French Native speaker (*M* = 3.1, *SD* = 1.4). This shows that regardless of the consolidation of L2 lexical learning, at the phonological and semantic levels, participants’ accent is perceived as foreign. Before cognate learning, participants perceived their accent in French as ‘discrete’ as opposed to ‘heavy’ or non-existent. The fact that participants did not find their accent heavy even before training, while raters perceived a heavy foreign accent following training indicates that L2 speakers and native-speaker listeners may have different perceptions regarding accent, (Yi et al., 2014). The reasons why this is so are difficult to tease apart, and may include motivation, awareness, expectancy related factors. However, given that the average age of participants to this study was 43 y/o, the results can be interpreted within the context of the critical period hypothesis (e.g., Long, 1990; Bongaerts et al., 1995; Birdsong, 1999; Singleton, 2003). Thus, the capacity to discriminate novel sounds is limited to a critical period, which ends between 6 and 12 months of age (Kuhl et al., 2003; Houston et al., 2007), and after which learners become less sensitive to differences between their productions and native accent (Long, 1990; Bongaerts et al., 1995; Birdsong, 1999; Singleton, 2003). Lack of awareness leads to persistence of foreign accent, regardless of high proficiency in naming, as reflected in this study by equivalent RT and ER in naming L1 and L2 Cognates.

The fMRI data showed significant activations in a number of motor processing and control areas. Specifically, the contrast (Cognate vs. Dido), showed a significant activation in the left Middle occipital gyrus, the left Lingual gyrus, the left Inferior frontal gyrus, the left Precentral gyrus, the left Inferior frontal gyrus, and the left, the right Middle occipital gyrus, the right Parahippocampal gyrus, and the right Cerebellar tonsil. These brain areas have been reported to sustain cognate processing, in previous work by our team, and others (De Bleser et al., 2003; Abutalebi, 2008; Raboyeau et al., 2010; Ghazi-Saidi et al., 2013; Marcotte and Ansaldo, 2014) and their role in motor (i.e., premotor cortex and supplementary motor areas; Raboyeau et al., 2010), attentional processing (i.e., anterior cingulate cortex, caudate nucleus, prefrontal cortex; Abutalebi, 2008), and word comprehension (i.e., anterior inferior temporal regions; De Bleser et al., 2003), has been consistently documented in healthy adult second language learners. Further, evidence from clinical data emphasizes the role of these areas in various lexical, motor and attentional processing. Interestingly, significant activations in a similar set of areas have been reported in studies on patients with FAS (Fridriksson et al., 2005; Poulin et al., 2007; Katz et al., 2012; Moreno-Torres et al., 2013; Tomasino et al., 2013), and damage to these areas in FAS patients (Kurowski et al., 1996; Mariën et al., 2006, 2009; Gurd and Coleman, 2006; Scott et al., 2006; Kuschmann et al., 2012). Finally, in a recent review, Carbary et al. (2000) conclude that FAS is typically associated to damage in the left pre-central gyrus and inferior frontal gyri, the basal ganglia the insula cortex, which are similar to the areas reported in the fMRI studies on healthy participants, specifically focusing on the bilingual lexicon through cognate processing (Carbary et al., 2000; Scott et al., 2006; Ghazi-Saidi et al., 2013; Marcotte and Ansaldo, 2014). Our results provide a supplementary source of evidence to the role of these areas with healthy participants learning a second language vocabulary.

As for the contrast (L2 vs. L1 Cognate Naming), it allowed highlighting the specific feature distinguishing between L2 andL1 cognate naming, and this corresponded to a single significant activation in the left insula. Thus, cognates share phonological and semantic features with mother tongue, but they differ in terms of prosody, intonation and articulation placement, all of which are essential components of accent. Accordingly, the significant activation in the insula reported here, specifically reflects the accent component of L2 picture naming. In the next section we discuss the specific role of the insula on accent processing.

### The Role of Insula in Accent Processing

Given its location in the brain, the role of insula in language processing was mostly examined in lesion studies. With the advancement of functional neuroimaging techniques, we have now access to literature that is not more than a decade old (Bamiou et al., 2003; Ackermann and Riecker, 2004; Menon and Uddin, 2010; Sterzer and Kleinschmidt, 2010; Damasio et al., 2013; Oh et al., 2014).

In line with Menon and Uddin (2010), the evidence reported in the present study stresses on the role of insula detecting salient events, specifically L2 accent patterns, which requires coupling attention, working memory, and motor planning for L2 word production. Moreover, in line with previous accounts on the role of the insula in vocal track manipulation for articulation and phonation (Ackermann and Riecker, 2004) and auditory processing (Bamiou et al., 2003), the significant activation of the insula observed in the present study can also be related to adjustments of the vocal track with the purpose of optimizing accent in L2.

From a broader cognitive perspective, Damasio et al. (2013), attribute to the insula a role in higher order cognitive and emotional processing, including subjective feelings from the body, and processing uncertainty. In line with this view, we believe that the activation of the insula in the context of persistent foreign accent can be related to higher order processes (Moyer, 2013) involved the ability to recognize, comprehend and integrate the segmental and suprasegmental levels of phonology with the purpose of achieving optimal word production. The insula’s role on higher order speech language processing can be related to its highly connected network, with speech, language, and executive function centers in the brain. (Oh et al., 2014), which facilitates the integration of a large variety of cognitive processes, ranging from motor to executive function that are put into play to achieve native-like pronunciation, even when target words are L2 cognates.

The significant activation of the left insula in the context of novel syllable processing has also been reported both with healthy populations (Chee et al., 2004) and in cases of AOS (Moser et al., 2009). Also with brain damaged populations, the insula’s role on accent processing has been documented in cases of FAS (Gurd and Coleman, 2006; Mariën and Verhoeven, 2007; Moreno-Torres et al., 2013; Tomasino et al., 2013), a finding that has lead a number of authors to hypothesize on the role of the insula in accent processing (Moonis et al., 1996; Carbary et al., 2000; Ackermann et al., 2004; Gurd and Coleman, 2006; Scott et al., 2006). The present study provides direct evidence to this hypothesis with healthy second language learners.

Moreover, the activation of the insula in the context of L2lexical learning has been reported in previous work by our team and others (De Bleser et al., 2003; Chee et al., 2004; Abutalebi, 2008; Ghazi-Saidi et al., 2013; Marcotte and Ansaldo, 2014), and its activation has been found to be positively correlated with decreased anterior cingulate and anterior frontal activation (Chee et al., 2004) Thus, Chee et al. (2004) argue that the insula plays a role in sensory-perceptual processing. Also, particularly relevant to the present study, is the evidence of the insula’s role on sub-vocal rehearsal of speech contrasts (Fiez et al., 1996; Smith et al., 1998; Callan et al., 2003, 2004), a finding that is in line with the DiVA Model (Guenther, 2006) which highlights the role of the insula in the selection of speech sound maps, thanks to its connection with the premotor and motor cortices.

Finally, from a more general cognitive perspective, the significant activation of the insula has also related to self-consciousness in the sense of agency, namely, experiencing oneself as being the cause of an action (Farrer and Frith, 2002). In the context of this study, the insula may as well be supporting awareness and regulation of accent features in L2 cognate production. Moreover, particularly involved in cognitive control (e.g., Menon and Uddin, 2010; Nelson et al., 2010; Tops and Boksem, 2011), the insula has been shown to be sensitive to salient events (Menon and Uddin, 2010); this finding together with its strong connectivity to the motor cortex (Ackermann and Riecker, 2004), argue in favor of a particularly important role of the insula when trying to attain native-like accent. The sensitivity to distinct accent features coupled with access to motor programming structures allows for feedback and feed-forward mechanisms at the core of L2 accent production.

## Conclusion

The results of the present study show that the production of a native-like accent remains challenging and cognitively effortful, even when L2 words share phonology and semantics with L1 equivalents, and despite the fact that vocabulary learning is consolidated. In line with clinical reports on FAS, and functional neuroimaging studies on accent production in healthy populations, the important role of the insula in accent processing may be related to a number of high order and highly accent specific processing features ranging from self awareness and monitoring, to vocal track control, and sub-vocal rehearsal of phonemic sequences.

The evidence provided by the present study is specific, as for the first the first time the role of the insula in accent processing is confirmed among healthy adults tested on a type of words whose only difference across mother tongue and second language is at the level of accent, namely cognates.

## Conflict of Interest Statement

The authors declare that the research was conducted in the absence of any commercial or financial relationships that could be construed as a potential conflict of interest.

## Footnotes

1 http://www.neurobs.com

2 www.fil.ion.ucl.ac.uk/spm/
