# Supplementary material for: How native-like can you possibly get: fMRI evidence for processing accent
Source: Front Hum Neurosci. 2015 Oct 30;9:587. doi: 10.3389/fnhum.2015.00587 (PMC4626569; doi:10.3389/fnhum.2015.00587)
Supplement: Supplementary file 1 [file Data_Sheet_1.DOCX]

Appendix 1. UNF’s MRI Screening form

|  | | | | | | **First Name** | | | | | | |
| --- | --- | --- | --- | --- | --- | --- | --- | --- | --- | --- | --- | --- |
|  | | | | | |  | | | | | | |
| **Date of Birth** : yyyy/mm/dd | | | | | | **Sex :** | F | | | | M | |
| **Height :** | ___________ m / ft | | | | | **Weight :** | ___________ kg / lbs | | | | | |
| Researcher (or) Project (or) Ethical Number : | | | | | | | | | | | | |
| **To ensure the safety of all individuals entering the Functional Neuroimaging Unit, it is of the utmost importance to complete the questionnaire correctly. All information contained in this document is confidential.** | | | | | | | | | | | | |
| 1. **Have you had any previous surgery ?** | | | | | | | | | | | | |
|  | | No | Yes | | If yes, indicate the type of surgery and date yyyy/mm/dd | | | | | | | |
| Head | |  |  | |  | | | | | | | |
| Heart or Chest | |  |  | |  | | | | | | | |
| Abdomen, pelvis | |  |  | |  | | | | | | | |
| Extremities (arm, hand) | |  |  | |  | | | | | | | |
| Extremities (leg, foot) | |  |  | |  | | | | | | | |
| Spine | |  |  | |  | | | | | | | |
| Eyes | |  |  | |  | | | | | | | |
| Others | |  |  | |  | | | | | | | |
|  | | | | | | | | | | | | |
| **2. Do you have any of the following ?** | | | | | | | | | | **No** | | **Yes** |
| Pace-maker? Epicardial wires | | | | | | | | | |  | |  |
| Aneurysm clips, Stent? | | | | | | | | | |  | |  |
| Filter or catheter in a blood vessel? | | | | | | | | | |  | |  |
| Artificial heart valve? | | | | | | | | | |  | |  |
| Cochlear implant ? Hearing aide? | | | | | | | | | |  | |  |
| Neurostimulator or Bone growth stimulator? | | | | | | | | | |  | |  |
| Metal foreign body (ex: bullets, fragments of shells, metal chips)? | | | | | | | | | |  | |  |
| Implanted insuline pumps? | | | | | | | | | |  | |  |
| Orthopedic implant (ex: crews, plate, pins)? | | | | | | | | | |  | |  |
| Tattoos or permanent make-up? | | | | | | | | | |  | |  |
| Piercing? | | | | | | | | | |  | |  |
| Implants magnetic or non-magnetic? | | | | | | | | | |  | |  |
| Diaphragm or IUD? | | | | | | | | | |  | |  |
| Dental work (ex. braces, caps, crowns, dentures)? | | | | | | | | | |  | |  |
| Ocular implants? | | | | | | | | | |  | |  |
| Transdermic patch (ex : nitroglycerine patch)? | | | | | | | | | |  | |  |
| Others*:* | | | | | | | | | |  | |  |
|  | | | | | | | | | | | | |
|  | | | | | | | | | | | | |
|  | | | | | | | | | | **No** | | **Yes** |
| **3. Are you pregnant or think you may be ?** | | | | | | | | | |  | |  |
| **4. Are you claustrophobic ?** | | | | | | | | | |  | |  |
| **5. Have you ever been injured by metal (ex : car accident, work accident, hunting, etc.) ?** | | | | | | | | | |  | |  |
| If yes, give a brief description of injury: | | | | | | | | | |  | |  |
|  | | | | | | | | | |  | |  |
| **6. Have you ever had an MRI ?** | | | | | | | | | |  | |  |
| **7. Have you ever been:** | | | | | | | | | |  | |  |
| Machinist ? | | | | | | | | | |  | |  |
| Welder ? | | | | | | | | | |  | |  |
| Heavy equipment operator ? | | | | | | | | | |  | |  |
| Metal worker ? | | | | | | | | | |  | |  |
| **8. Do you have any respiratory or motor conditions ?** | | | | | | | | | |  | |  |
|  | | | | | | | | | | | | |
| The MRI study has been explained to me. The safety measures were explained to me and all my questions were answered. All the questions above were answered accurately to the best of my knowledge, and I consent to participate in a MRI study. | | | | | | | | | | | | |
|  | | | | | | | | | | | | |
|  | | | | | | | | | | | | |
| **PARTICIPANT:** | | | | | | | | | | | | |
|  | | |  |  | | | |  | yyyy/mm/dd | | | |
| **Print letters** | | |  | **Signature** | | | |  | **Date** | | | |
|  | | |  |  | | | | |  | | | |
|  | | |  |  | | | | |  | | | |
| **RESEARCHER*/*TECHNOLOGIST/ MRI OPERATOR*:*** | | | | | | | | | | | | |
|  | | |  |  | | | |  | yyyy/mm/dd | | | |
| **Print letters** | | |  | **Signature** | | | |  | **Date** | | | |
|  | | |  |  | | | | |  | | | |
|  | | | | | | | | | | | | |

Appendix 2. Questionnaire and the scale filled up bu Canadian French Native raters.

**Questionnaire sur antecedents linguistiques**

Nom, prénom: ___________________________________________________________

Identification de l’intervenant: _______________________________________________

Date: ________________ Âge: ______ Lieu de naissance : _______________________

Province et villeoùvousavezgrandi: __________________________________________

Oùvivez-vousprésentement? : _________________________________________________

Avez-vous des problèmesd’ouïe? (Encercler) OUI NON

Nombred’annéesd’éducation: _____________________

S.V.P listerd’autreslanguesquevousconnaissez et votreniveau de compétence pour chaque :

________________________________________________________________________________________________________________________________________________________________________________________________________________________________________________________________________________________________________________________

Appendix 2:

Veuillezencercler la cote quevousdonnez à chacun des participants.

Cotezsurl’échelle de 1 à 9, 1 étanttrèsétranger et 9 étant quasi natif.

| **Participants** | **Très étranger Natif** |
| --- | --- |
| **P1** | 1 2 3 4 5 6 7 8 9 |
| **P2** | 1 2 3 4 5 6 7 8 9 |
| **P3** | 1 2 3 4 5 6 7 8 9 |
| **P4** | 1 2 3 4 5 6 7 8 9 |
| **P5** | 1 2 3 4 5 6 7 8 9 |
| **P6** | 1 2 3 4 5 6 7 8 9 |
| **P7** | 1 2 3 4 5 6 7 8 9 |
| **P8** | 1 2 3 4 5 6 7 8 9 |
| **P9** | 1 2 3 4 5 6 7 8 9 |
| **P10** | 1 2 3 4 5 6 7 8 9 |
| **P11** | 1 2 3 4 5 6 7 8 9 |
| **P12** | 1 2 3 4 5 6 7 8 9 |
